# Weekly Text Messages to Support Adherence to Oral Pre-Exposure Prophylaxis (PrEP) Among Gay, Bisexual, and Other Cisgender Men Who Have Sex With Men (MSM) and Transgender Women: Pilot Randomized Controlled Trial Nested in PrEP Brasil Study

**DOI:** 10.2196/72360

**Published:** 2025-07-18

**Authors:** Luana Monteiro Spindola Marins, Thiago Silva Torres, Ronaldo Ismerio Moreira, Iuri Costa Leite, Marcelo Cunha, Brenda Hoagland, Lucilene Araujo Freitas, Debora Castanheira, Carolina Coutinho, Emilia Moreira Jalil, Mayara Secco Torres Silva, Jose Valdez Madruga, Beatriz Grinsztejn, Valdilea Gonçalves Veloso

**Affiliations:** 1 Instituto Nacional de Infectologia Evandro Chagas, Fundação Oswaldo Cruz, Av Brasil 4365 Manguinhos, Rio de Janeiro, 21040-360, Brazil, 55 2138659623; 2 Escola Nacional de Saúde Pública Sérgio Arouca, Fundação Oswaldo Cruz, Rio de Janeiro, Brazil; 3 Centro de Referência e Treinamento DST/AIDS, São Paulo, Brazil

**Keywords:** HIV, mHealth, digital health, Latin America, LGBTQIAPN+

## Abstract

**Background:**

Mobile phones have become popular tools to support public health interventions (mobile health [mHealth]). Text messaging, including SMS, is a simple, low-cost approach for health communication to a large population and offers valuable tools in improving health outcomes. Despite the global advances in HIV treatment and prevention, the HIV epidemic continues to disproportionately affect certain populations such as men who have sex with men (MSM) and transgender women, including in Latin America.

**Objective:**

This study aimed to evaluate the effectiveness of SMS text messaging in improving adherence after 1-year provision of oral pre-exposure prophylaxis (PrEP) among MSM and transgender women in Brazil.

**Methods:**

Pilot randomized controlled trial nested in the PrEP Brasil study, a prospective, multicenter, open-label implementation study assessing PrEP delivery in the context of the Brazilian Public Health System. Those who agreed to participate in the pilot trial were randomized 1:1 to standard-of-care (SOC) or intervention (SMS text messaging) arm. SMS text messages were launched fully automated weekly to participants for 48 weeks. Adequate adherence to PrEP has been defined as having (1) tenofovir-diphosphate concentration of ≥700 fmol/punch, (2) medication possession ratio of ≥1.07, (3) pill count of ≥90.1%, and (4) self-report (structured questionnaire) of ≥99.9%. Adequate adherence outcomes at week 48 were compared between arms (SMS text messaging vs SOC) using univariate logistic regression. Comparisons were also performed for young MSM aged 18‐24 years and transgender women.

**Results:**

From 450 participants enrolled on PrEP Brasil, 417 (92.7%) were randomized to the pilot trial: 210 to SOC and 207 to SMS arm. Until week 48, participants received a total of 14,099 SMS with the text message: “Are you okay?,” and 6959 (49.4%) messages were replied. Of these, the vast majority replied “Yes” (6762/6959, 97.2%). A total of 347 (83.2%) participants completed the study with no difference between arms for the 4 adherence outcomes. Conversely, young MSM who received SMS text messages had 2.50 increased odds of having adequate PrEP adherence measured by medication possession ratio (*P*=.05). Most participants found SMS text messaging very useful or useful (127/167, 76.0%) and would recommend SMS text messaging as a support strategy for persons using PrEP (134/167, 80.2%). Most participants think that SMS text messaging should be offered to all persons using PrEP (129/167, 77.2%), and 16.2% (27/167) think that SMS text messaging should be offered only to those persons using PrEP with adherence problems. Weekly messages were found adequate by 80.2% (134/167).

**Conclusions:**

SMS text messaging intervention improved adequate PrEP adherence among young MSM and can be a useful tool for PrEP coverage and persistence. Future interventions using other mHealth tools such as WhatsApp messages and apps tailored to support PrEP adherence should be evaluated among MSM and transgender women in Brazil.

## Introduction

Despite the global advances in HIV treatment and prevention, the HIV epidemic continues to disproportionately affect certain key populations such as gay, bisexual, and other men who have sex with men (MSM), and transgender women. Estimated proportions of new adult infections in 2010 and 2022 increased for MSM and transgender women across all regions [1]. In Latin America, the number of new HIV cases has increased between 2010 and 2023, highlighting persistent challenges in controlling the epidemic [2]. In Brazil, recent data indicate an increasing HIV prevalence among young MSM aged 18‐24 years [3,4]. The estimated annualized HIV incidence among MSM and transgender women in the country was 2.62% (95% CI 1.78‐3.43), being higher among young persons aged 18‐24 years (3.48%, 95% CI 1.99‐4.94) [5]. Recently, HIV prevalence was estimated at 10% in a respondent-driven sampling–based study among young MSM aged 18‐24 years in Rio de Janeiro, Brazil [6].

The safety and efficacy of oral pre-exposure prophylaxis (PrEP) with emtricitabine plus tenofovir disoproxil fumarate in reducing the risk of HIV acquisition [7-10] and the relationship between PrEP adherence and protective efficacy have been well demonstrated [11-13]. During the ImPrEP study, the largest PrEP implementation study in Latin America, HIV incidence was higher among those with lower adherence measured by medication possession ratio (MPR) [14]. Additionally, adherence was lower among younger participants (aged 18‐24 years), transgender women, persons self-identifying as Black, and those with lower education [14,15].

The rapid growth of mobile technologies and their popularity worldwide led to solutions to address problems within health care systems (mobile health [mHealth]). Thus, mobile phones have become popular tools to support medical or public health, offering new possibilities for diagnosis, treatment, interventions, mHealth apps, and training [16]. Text messaging, including SMS, is a simple, low-cost approach for health communication to a large population and offers valuable tools in improving health care outcomes. In an umbrella review of 34 meta-analyses, representing 235 randomized controlled trials across 52 countries and 48,957 participants, text messages were effective for populations with chronic diseases, including for medication adherence among people living with HIV [17]. In Brazil, results from a randomized controlled trial indicated that SMS text messaging improved antiretroviral therapy adherence among cisgender women living with HIV [18]. In this sense, there is a broad interest in the scientific development and use of mHealth in HIV prevention [19].

Although adherence is a requisite to PrEP protection, few studies evaluating mHealth interventions to support PrEP adherence were conducted in Latin America [20]. Qualitative findings from a study conducted among young MSM aged 18‐24 years in Brazil have shown high acceptability of weekly text messages to improve PrEP adherence [21]. In this sense, the aim of this study was to evaluate the effectiveness of weekly SMS text messaging in improving adherence after 1-year provision of oral PrEP among MSM and transgender women in Brazil.

## Methods

### Study Design

Randomized controlled pilot trial (SMS substudy) nested in PrEP Brasil study, a prospective, multicenter, 48-week open-label implementation study assessing PrEP delivery for MSM and transgender women at higher vulnerability for HIV in the context of the Brazilian Public Health System (Sistema Único de Saúde). Details of the PrEP Brasil study design and results were published elsewhere [22-26]. Briefly, participants were recruited in 3 sites in Rio de Janeiro and São Paulo, Brazil. They completed a structured sociobehavioral questionnaire and received 1 bottle containing 30 pills of tenofovir disoproxil fumarate for PrEP at enrollment. Subsequently, participants received a sufficient supply of pills for daily use at weeks 4, 12, 24, and 36. Study pharmacists instructed the participants to take 1 daily pill and to return remaining PrEP pills in the original bottle to the pharmacy for accountability. At each study visit, the pharmacists and psychologists provided adherence support and counseling as standard of care (SOC).

The SMS substudy was offered to all participants enrolled in the PrEP Brasil study. Individuals were eligible if they had an SMS text messaging–capable mobile phone and were willing to use this phone for receiving study text messages. Eligible participants were randomized 1:1 to SOC arm or SOC plus weekly SMS text messages (SMS arm). The data manager used block randomization (n=10) to ensure an unbiased allocation into the 2 arms. The data manager prepared individual assignment cards that indicated the group allocation for each eligible participant. Each assignment card was placed in a sealed opaque envelope to conceal the group allocation. Then, each envelope was assigned a unique identification number corresponding to its sequence in the randomization process. During enrollment, site study coordinators accessed the next numbered envelope in sequence to assign each participant’s arm. Pharmacists and psychologists providing SOC were blinded after the study arm assignment. All participants received the intended care, regardless of allocated arm. The SMS substudy was approved by the institutional review boards at Instituto Nacional de Infectologia Evandro Chagas (INI-Fiocruz), Universidade de São Paulo, and Centro de Referência e Treinamento DST/AIDS de São Paulo. All participants provided written consent to participate in the substudy. This study was registered at clinicaltrial.gov, NCT01989611 (November 21, 2013) and follows CONSORT-EHEALTH (Consolidated Standards of Reporting Trials of Electronic and Mobile Health Applications and Online Telehealth) guidelines (the CONSORT-EHEALTH checklist is provided in Checklist 1).

### Sample Size

All participants from the PrEP Brasil study were invited to the SMS substudy, with no prior sample size calculation. A systematic review from 2012 reported that the mean proportion of participants with oral PrEP adequate adherence was 87%, ranging from 69% to 97% [27]. Based on this, a sample size of 153 participants per arm was deemed sufficient to provide 90% power to detect a 10-percentage-point difference in adherence rates, using a 2-sided test at the 5% significance level. In the TAPIR study, a randomized controlled trial assessing oral PrEP adherence at week 48 compared daily SMS text messaging intervention with SOC. Approximately 200 participants per arm were enrolled, with 324 participants retained at week 48. This sample size was calculated to detect a 15% difference in adherence between the arms, assuming 68% adherence in the SOC group, with 94% power, and a 2-sided α value of .05 [28].

### SMS Text Messaging and Messaging System Platform

INI-Fiocruz Information Technology staff developed an automated password-protected messaging system platform. This platform had technical safeguards to protect participant information and confidentiality as only authorized study staff (including the study coordinator) using a personal username and password had access. The study coordinator included the participants’ mobile numbers allocated to the SMS arm into the platform. Through the password-protected platform database, the authorized staff could supervise the SMS text messages exchanged with the participants. The platform access and integrity could be monitored and audited only under authorized staff supervision.

All participants allocated to the SMS arm received a morning weekly SMS text message (“Are you okay?” in the local language) from the automated platform after baseline visit, starting on week 1 until week 47. The SMS text message content (“Are you okay?”) was purposefully chosen for its neutral tone, ensuring that it did not disclose participants’ involvement in the study or their engagement with HIV prevention services. Our primary objective was to safeguard participant confidentiality and reduce the risk of unintended disclosure to third parties, which could potentially lead to negative social consequences [29,30].

**Figure 1.**
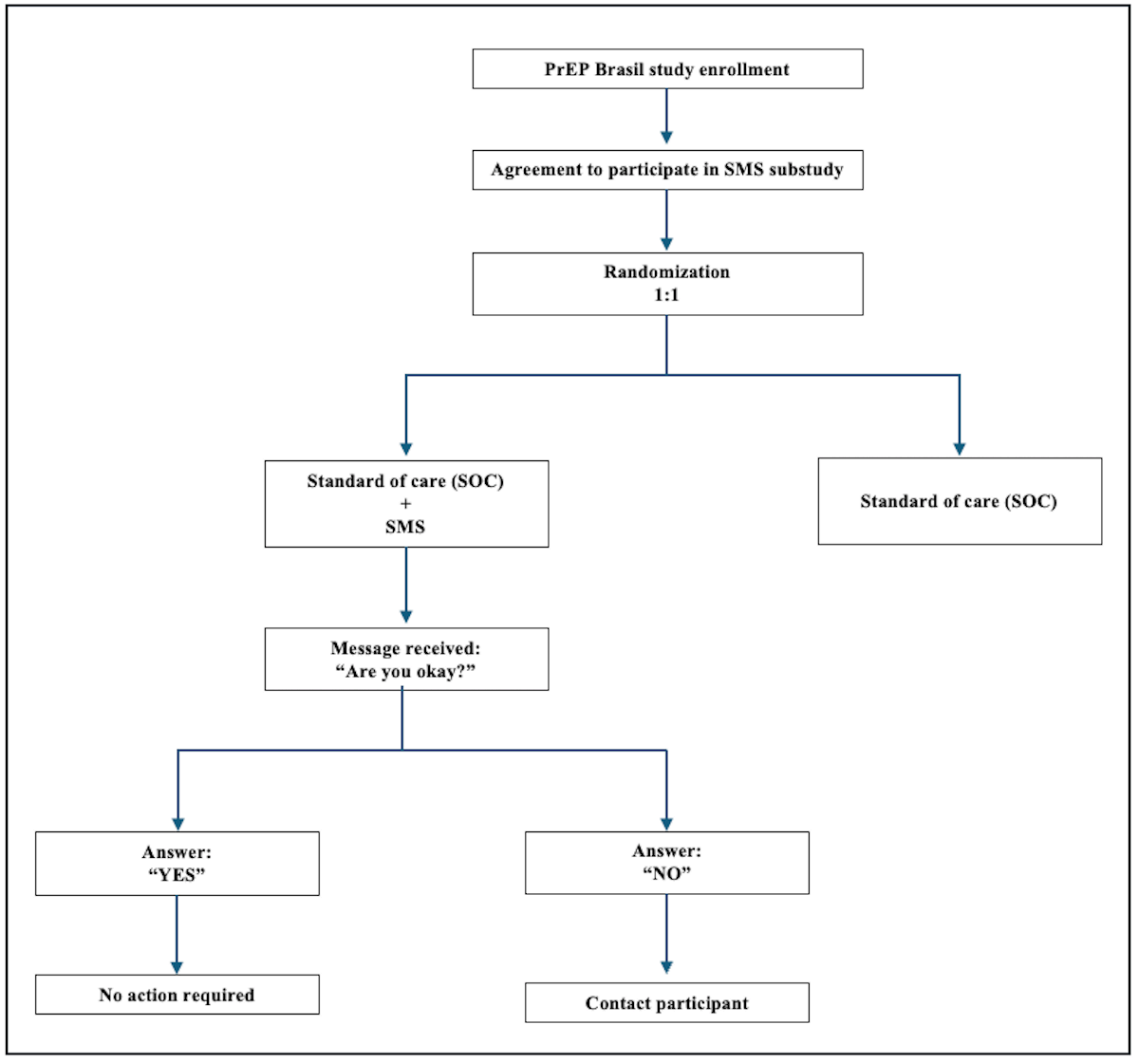
PrEP Brasil SMS substudy design flowchart. PrEP: pre-exposure prophylaxis.

Participants were instructed to respond “Yes” or “No.” Those who answered “Yes” received an acknowledgment “Thank you” text message. The study coordinator contacted all participants replying “No” within 48 hours and provided adherence support. The platform automatically sent a second SMS text message at night for the participants not responding to the initial SMS text message. The study coordinator contacted by phone call all participants not responding for 3 consecutive weeks (Figure 1). SMS text message response was charged to participants according to their mobile plan; participants did not receive a budget from the study. We instructed the participants not to use SMS text messages for medical emergencies. Instead, they were instructed to contact the study site by phone or proceed immediately to the site.

### Adherence Outcomes

Adherence was measured at week 48 in the PrEP Brasil study using four different methods: (1) tenofovir-diphosphate (TFV-DP) levels, (2) MPR, (3) pill count, and (4) self-report. TFV-DP levels were measured on blood from dried blood spots, using liquid chromatography-mass spectrometry tandem mass spectrometry at the University of Colorado Antiviral Pharmacology Laboratory using standard procedures [13]. In a previous analysis using data from PrEP Brasil, we found concordance between TFV-DP levels and indirect adherence measures (MPR, pill count, and self-report) [24]. Considering these prior findings, we incorporated all 4 adherence measurement methods in our analysis, acknowledging that indirect methods are more affordable and accessible, making them more feasible for real-world implementation. MPR reflects the days the participant is “covered” by the study medication. It is calculated by the ratio of the number of pills dispensed at the prior visit and the number of days between that visit and the week 48 visit. For participants who missed the week 36 visit, MPR and pill count at week 48 were calculated based on data from their most recent attended visit. MPR values of 1.00 or greater indicate 100% PrEP coverage, and values below 1.00 reflect that the participant was not covered by PrEP during all the days between week 48 and the prior visit [14,24]. Pill count was calculated by the number of pills dispensed at the prior visit minus the number of pills returned at week 48, divided by the number of days between the 2 visits. Finally, self-report was assessed using 1 question from a structured questionnaire (“On average for how many days did you forget to take your PrEP pills in the previous 30 days?") administered by study pharmacists. The iPrEX Open-label study demonstrated that TFV-DP concentration of ≥700 fmol/punch, corresponding to ≥4 doses per week, was associated with a 100% (95% CI 86%-100%) reduction in HIV transmission [13]. In addition, based on our group’s previous exploratory analysis to determine the cutoff points for each indirect adherence measure [24], we defined adequate PrEP adherence at week 48 as (1) TFV-DP concentration of ≥700 fmol/punch, (2) MPR of ≥1.07, (3) pill count of ≥90.1%, and (4) self-report of ≥99.9%.

### SMS Text Message Acceptability Assessment

Participants allocated to SMS arm completed a structured questionnaire about SMS text message acceptability at week 48. Participants responded to predefined multiple-choice questions regarding usefulness of SMS text message service, frequency of SMS text messages, problems receiving or sending SMS text message, and platform preference (email or SMS text message). The responses were summarized as frequencies and percentages.

### Statistical Analysis

We conducted initial descriptive analyses to compare baseline characteristics, the randomization of participants, and the distribution of variables. PrEP adherence outcomes at week 48 were compared between arms (SMS vs SOC) using univariate logistic regression (odds ratio and 95% CI). We also compared PrEP adherence outcomes at week 48 between arms for 2 groups: young MSM (aged 18‐24 years) and transgender women. Analyses were conducted using SAS (version 9.4; SAS Institute).

### Ethical Considerations

INI Evandro Chagas-FIOCRUZ institutional review board has approved this study (no. CAAE08405912.9.1001.5262 at ‘‘Plataforma Brasil’’) and all study participants have signed an informed consent form. Institutional review boards at CRT-AIDS and Universidade de São Paulo also approved the study after first approval had been granted at Fiocruz.

## Results

**Figure 2.**
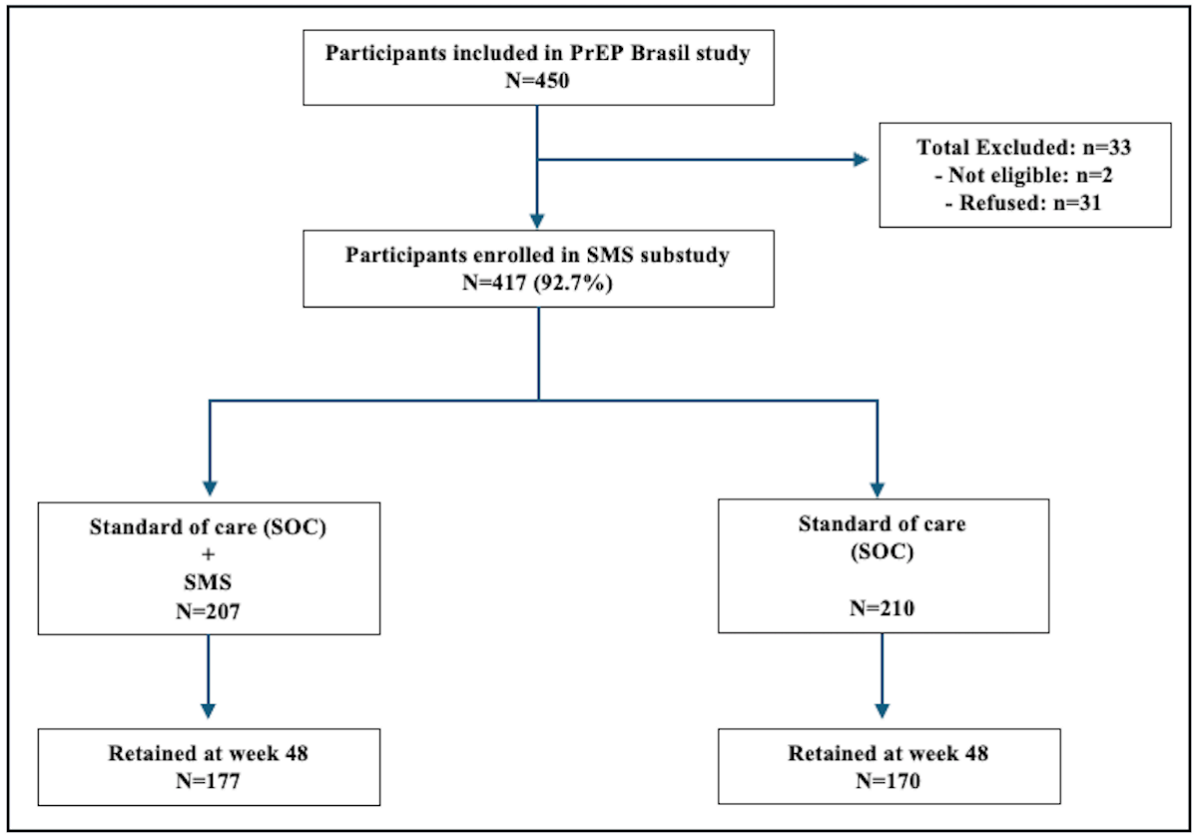
CONSORT (Consolidated Standards of Reporting Trials) flow diagram of the PrEP Brasil SMS substudy. PrEP: pre-exposure prophylaxis.

From April 2014 to July 2016, 450 participants were enrolled and followed for 48 weeks in the PrEP Brasil study. Of these, 31 (6.9%) participants refused to participate in the SMS substudy, and 2 (0.4%) were ineligible (no personal mobile phone to receive SMS text messages). Thus, 417 participants were included and randomized in the SMS substudy to SMS arm (N=207) and SOC arm (N=210) (Figure 2).

Baseline characteristics of participants included in the SMS substudy (N=417) according to arm are described in Table 1. Overall, the median age was 30 years (IQR 25‐35 years); 102 (24.5%) participants were aged 18‐24 years. The majority self-identified as cisgender men (392, 94.0%) and 25 (6.0%) as transgender women. More than half (246, 59.0%) were from São Paulo and White (224, 54.4%), and 307 (73.6%) completed 12 years or more of schooling. Regarding sexual behavior 3 months prior to the baseline visit, 184 (44.1%) participants reported condomless receptive anal sex, and 205 (49.2%) participants reported sex with partners living with HIV. A total of 82 (19.7%) participants have at least 1 sexually transmitted infection diagnosed at baseline. Most participants reported binge drinking (250, 60.0%), while 79 (18.9%) participants reported use of stimulants.

**Table 1.** Baseline characteristics of participants enrolled in the PrEP Brasil SMS substudy according to arm (SMS text messaging vs standard of care).

Characteristics	SMS	SOC^a^	Total
Total, n (%)	207 (49.6)	210 (50.4)	417 (100)
Site location, n (%)			
Rio de Janeiro	85 (41.1)	86 (41.0)	171 (41.0)
São Paulo	122 (58.9)	124 (59.0)	246 (59.0)
Age (years), n (%)			
18‐24	60 (29.0)	42 (20.0)	102 (24.5)
25‐35	90 (43.5)	111 (52.9)	201 (48.2)
>35	57 (27.5)	57 (27.1)	114 (27.3)
Gender, n (%)			
Cisgender men	195 (94,2)	197 (93,8)	392 (94.0)
Transgender women	12 (5.8)	13 (6.2)	25 (6.0)
Race, n (%)			
White	109 (53.4)	115 (55.3)	224 (54.4)
Black	23 (11.3)	29 (13.9)	52 (12.6)
*Pardo* or Mixed	72 (35.3)	64 (30.8)	136 (33.0)
Schooling (years), n (%)			
<12	56 (27.1)	54 (25.7)	110 (26.4)
≥12	151 (72.9)	156 (74.3)	307 (73.6)
Condomless receptive anal sex, n (%)^b^			
Yes	89 (43.0)	95 45.2)	184 (44.1)
No	118 (57.0)	115 (54.8)	233 (55.9)
Sex with partners living with HIV, n (%)^b^			
Yes	104 (50.2)	101 (48.1)	205 (49.2)
No	103 (49.8)	109 (51.9)	212 (50.8)
STI^c^ diagnosis, n (%)^d^			
Yes	39 (18.8)	43 (20.5)	82 (19.7)
No	168 (81.2)	167 (79.5)	335 (80.3)
Binge drinking, n (%)^b^^,^^e^			
Yes	127 (61.4)	123 (58.6)	250 (60.0)
No	80 (38.6)	87 (41.4)	167 (40.0)
Use of stimulants, n (%)^b^^,^^f^			
Yes	38 (18.4)	41 (19.5)	79 (18.9)
No	169 (81.6)	169 (80.5)	338 (81.1)

a SOC: standard of care.

b Prior 3 months before baseline visit.

c STI: sexually transmitted infection.

d Any positive laboratory diagnosis for syphilis, gonorrhea, or chlamydia at screening.

e Five or more drinks in a couple of hours.

f Cocaine, crack, amphetamines, or club drugs (ecstasy, LSD, ketamine, and GHB).

**Figure 3.**
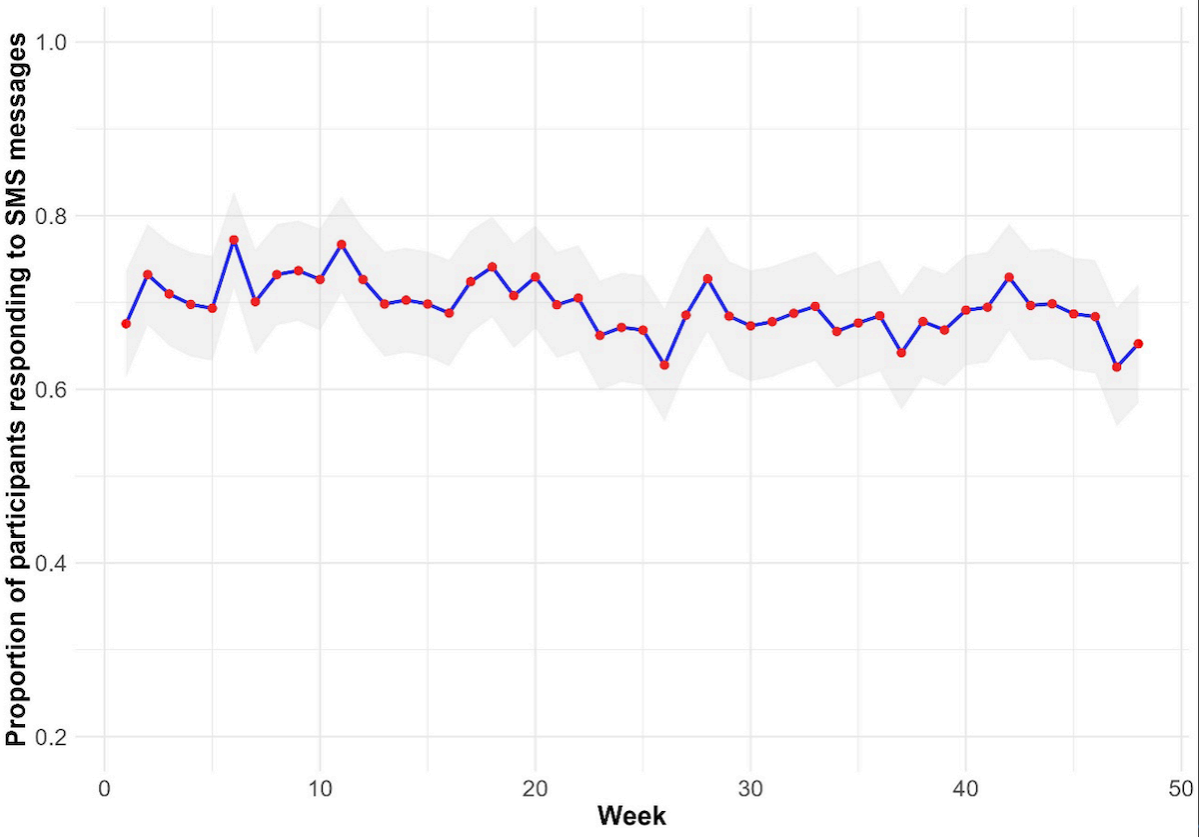
Proportion of participants replying to weekly SMS text messages over 48 weeks (PrEP Brasil SMS substudy).

Until week 48, participants received a total of 14,099 SMS with the text message: “Are you okay?,” and 6959 (49.4%) messages were replied (Table 2). Of these, the majority replied “Yes” (6762/6959, 97.2%) and 169 (2.4%) replied “No.” Most participants answering “No” reported health-related problems and were referred for an unscheduled visit (108/169, 63.9%). We performed 776 phone contact attempts for the SMS text message not replied to after 3 consecutive weeks. Of these, 268 (34.5%) were successful, and the main reason reported was an inability to receive the messages (56, 20.9%). The proportion of participants responding to SMS text messages remained relatively consistent over the weeks, with response rates fluctuating slightly around 70% (Figure 3).

**Table 2.** Description of SMS text messages delivered during the PrEP Brasil SMS substudy.

	SMS text message
Replied SMS text message (n=14,099), n (%)
Yes	6959 (49.4)
No	7140 (50.6)
Are you okay? (n=6959), n (%)	
Yes	6762 (97.2)
No	169 (2.4)
Nonstandard answer	28 (0.4)
Reasons for replying “no” (n=169), n (%)	
Health-related problems; participant referred for an unscheduled visit	108 (63.9)
Personal problems	21 (12.4)
Needing some type of guidance	3 (1.8)
Not using PrEP^a^; referred to study termination visit	2 (1.2)
Did not answer	35 (20.7)
Successful phone contacts (participants not replying for 3 consecutive weeks) (n=776), n (%)	
Yes	268 (34.5)
No	508 (65.5)
Reasons for not replying SMS text message (n=268), n (%)	
Inability to receive the messages	56 (20.9)
Unwillingness to answer	44 (16.4)
No available credit on prepaid phone	37 (13.8)
Mobile phone device problems	34 (12.7)
Personal problems	30 (11.2)
Difficulties replying to messages	29 (10.8)
Desire to withdraw the study	15 (5.6)
Had a scheduled visit close by	13 (4.9)
PrEP Brasil study discontinuation	7 (2.6)
Problems with daily routine	2 (0.7)
Health-related problems	1 (0.4)

a PrEP: pre-exposure prophylaxis.

A total of 347 (417, 83.2%) participants completed the study (week 48), with no difference between arms (SMS text messaging: 177 vs SOC: 170; *P*=.21). Out of 347 participants, 12 (3.5%) participants did not attend the week 36 visit; hence, data from their most recent prior visit were used for MPR calculation. Considering all participants (overall), the proportion of participants with adequate PrEP adherence at week 48 did not differ between arms for all evaluated outcomes: TFV-DP levels, MPR, self-report, and pill count (Table 3). For young MSM, we found a difference between arms only for MPR: participants at the SMS arm had 2.50 increased odds (95% CI 1.01‐6.17) of having adequate PrEP adherence measured by MPR than those at the SOC arm (*P*=.05). For transgender women, we found no difference between arms for all PrEP adherence outcomes.

**Table 3.** Proportion of participants with adequate pre-exposure prophylaxis adherence at week 48 according to allocated arm (SMS text messaging vs standard of care) and comparison between arms in the PrEP Brasil SMS substudy.

	SMS, n (%)	SOC^a^, n (%)	OR^b^ (95% CI)	*P *value
Overall (N=347)				
TFV-DP^c^ levels	177 (75.1)	170 (73.5)	1.09 (0.67‐1.76)	.73
MPR^d^	177 (63.8)	170 (60.0)	1.18 (0.76‐1.82)	.46
Self-report	166 (57.8)	155 (60.0)	0.91 (0.59‐1.43)	.69
Pill count	165 (67.9)	156 (68.0)	1.00 (0.62‐1.59)	.99
Young MSM^e^ (18‐24 years) (n=81)				
TFV-DP levels	46 (80.4)	35 (68.6)	1.88 (0.68‐5.22)	.22
MPR	46 (65.2)	35 (42.9)	2.50 (1.01‐6.17)	.05
Self-report	40 (52.5)	28 (53.6)	0.96 (0.36‐2.52)	.93
Pill count	42 (66.7)	28 (57.1)	1.50 (0.56‐4.02)	.42
Transgender women (n=21)				
TFV-DP levels	11 (45.4)	10 (80.0)	0.23 (0.02‐1.94)	.24
MPR	11 (45.4)	10 (70.0)	0.38 (0.04‐2.89)	.49
Self-report	10 (20.0)	10 (50.0)	0.27 (0.02‐2.47)	.35
Pill count	10 (20.0)	10 (50.0)	0.27 (0.02‐2.47)	.35

a SOC: standard of care.

b OR: odds ratio:

c TFV-DP: tenofovir-diphosphate.

d MPR: medication possession ratio.

e MSM: men who have sex with men.

A total of 167 participants completed the SMS text message acceptability assessment (Table 4). Most participants found SMS text messaging service very useful or useful (127/167, 76.0%) and would recommend SMS text messaging as a support strategy for persons using PrEP (134/167, 80.2%). Most participants think that SMS text messaging should be offered to all persons using PrEP (129/167, 77.2%), and 16.2% (27/167) of participants think that SMS text messaging should be offered only to those persons using PrEP with adherence problems. Weekly messages were found adequate by 80.2% (134/167). Only 22.8% (38/167) had problems sending or receiving SMS text messages, and the most common reason was lack of prepaid credit (8/38, 21.1%). Only 11.4% (140/167) of participants would prefer to receive support messages via email rather than SMS text messaging.

**Table 4.** SMS text message acceptability assessment in the PrEP Brasil SMS substudy (N=167).

	Values, n (%)
Overall, how would you rate the usefulness of the SMS text messaging service?	
Very useful	71 (42.5)
Useful	56 (33.5)
Somewhat useful	26 (15.6)
Not useful	12 (7.2)
Missing	2 (1.2)
Which statement best describes how you feel?	
SMS text messaging should be offered to all persons using PrEP^a^	129 (77.2)
SMS text messaging should be offered only to those persons using PrEP with adherence problems	27 (16.2)
SMS text messaging should not be offered as PrEP adherence support	8 (4.8)
Missing	3 (1.8)
Would you recommend using SMS text messaging as a support strategy for persons using PrEP?	
Yes	134 (80.2)
No	18 (10.8)
Not sure	14 (8.4)
Missing	1 (0.6)
What did you think about the frequency of the weekly messages?	
Too frequent (too many messages)	9 (5.4)
Adequate	134 (80.2)
Insufficient (very few messages)	22 (13.2)
Missing	2 (1.2)
Did you have any problems sending or receiving SMS text messages during the study?	
Yes	38 (22.8)
No	127 (76.0)
Missing	2 (1.2)
*If yes, please describe (N=38*)	
I ran out of prepaid credit	8 (21.1)
I lost my phone	4 (10.5)
My phone was stolen	5 (13.2)
I did not have access to my phone	4 (10.5)
Other	13 (34.2)
Missing	4 (10.5)
How many times did you seek support from the study site staff using SMS text message?	
Never	144 (86.2)
Once	16 (9.6)
Twice	3 (1.8)
3 or more times	0 (0.0)
Missing	4 (2.4)
Would you prefer to receive support messages via email rather than SMS text messaging?	
Yes	19 (11.4)
No	140 (83.8)
Not sure	5 (3.0)
Missing	3 (1.8)

a PrEP: pre-exposure prophylaxis.

## Discussion

### Principal Findings

PrEP Brasil SMS substudy was the first effort to develop a mHealth intervention to improve oral PrEP adherence among MSM and transgender women in Latin America. Our results show that weekly SMS text messages were highly acceptable to support PrEP use, but they were not effective in increasing adequate PrEP adherence at week 48 among MSM and transgender women in Brazil. This could be partially explained by the preference of WhatsApp over SMS text messages in the country, including among MSM and transgender women [21,31]. In fact, approximately 96% of the Brazilian population uses WhatsApp [32], and the platform is considered more accessible and cost-effective than traditional SMS text messaging [33]. However, SMS text messaging was effective in improving adequate PrEP adherence measured by MPR among young MSM. Adequate adherence by MPR means that the participant has received enough pills to take daily oral PrEP, being “covered” by PrEP pills during all days between the study visits. Adequate MPR also indicates engagement with the service, as participants returned for PrEP prescriptions on time. In this sense, our study indicates that SMS text messaging can be effective to increase PrEP coverage and persistence among young MSM.

In a previous analysis conducted in the PrEP Brasil study, we found that SMS text messaging increased adequate PrEP adherence measured by TDF-DP levels among young participants [22]. Some differences may explain these different results. First, the current analysis included only those participants enrolled in the SMS substudy. As such, the lack of significant statistical difference may have occurred due to a lower sample size. Second, the current statistical analysis compared outcomes at the 48-week visit, while the prior considered adherence levels across all follow-up visits. Finally, in the current analysis, we evaluated adherence stratified for young participants self-identified as cisgender men, while in the previous analysis, we grouped together all youth (both cisgender men and transgender women).

Efficacy of SMS text message reminders among young MSM in our study may be linked to their higher familiarity with technology and digital communication [34]. Younger individuals tend to be more engaged with mobile devices and more open to digital health interventions [35]. Youth may also be more used to receiving and responding to automated messages, increasing the chance that these messages will support PrEP use and service engagement. This suggests that tailoring adherence interventions to the preferred communication channels of different age groups could improve their effectiveness.

Our results provide evidence that text message interventions should be used to improve adherence outcomes, such as PrEP coverage and persistence, among young MSM, a population group with lower adherence compared with older counterparts [14]. Youth-tailored multicomponent mHealth intervention could be developed considering the Brazilian context. For instance, PrEPmate, an mHealth intervention grounded in the information, motivation, and behavioral theory of behavior, increased PrEP adherence among young MSM and transgender women in the United States [36].

Other studies evaluating reminder text messages to improve adherence have shown conflicting results across different populations and settings. Fuchs and colleagues [30] demonstrated that a weekly bidirectional SMS text messaging was feasible, highly acceptable, and efficacious to improve PrEP adherence. However, SMS text messaging reminders were ineffective in promoting PrEP adherence among MSM and transgender women in the United States [28] and cisgender women aged 18‐24 years in Kenya [37]. These conflicting findings underscore the importance of considering not only the structure of the intervention but also the sociocultural and technological context in which it is implemented. The frequency and familiarity of SMS text messaging usage differ widely across countries. In some settings—such as Brazil—alternative messaging platforms such as WhatsApp may be more prevalent and better received.

Other interventions alone or combined should be evaluated among MSM and transgender women in Brazil, including text messaging reminders using WhatsApp or apps, alarms, video games, cognitive behavioral therapies, motivational interviewing and counseling, support groups, or forums and peer navigation [20]. Additionally, it is important to evaluate the content of these messages, as several studies indicate that participants prefer messages that go beyond simple reminders, offering more engaging and meaningful content and the optimal frequency of reminders [30,38-40].

Although only daily oral PrEP was recommended in Brazil during the study conduction, event-driven oral PrEP (ED-PrEP) is now part of national guidelines [41]. ED-PrEP will affect how we interpret indirect adherence assessments (eg, pill counts or MPR) as decisions on PrEP regimen and PrEP pill taking will depend on individuals’ demands and choices based on HIV-perceived risk and sexual behavior. In this sense, adequate pill availability is essential for effective daily oral PrEP or ED-PrEP, and SMS text messaging can be a useful technology to prevent pill shortage.

### Strengths and Limitations

The major strength of this study is the randomized clinical trial design with allocation concealment. The demographics and baseline characteristics of the study groups were well balanced, and there was no significant difference in sample characteristics between intervention arms and, as such, less affected by selection or sampling bias. Although this was not a blinded study, 2 main outcomes (TFV-DP level and MPR) are not liable to human interference [42,43]. In addition, this was a low-cost intervention as we could program the SMS text messaging platform to submit a large number of text messages to different mobile phone numbers simultaneously.

This study has limitations. First, our analysis was cross-sectional and restricted to adherence measured at week 48, as dried blood spots specimens for TFV-DP levels were collected from all participants exclusively at this visit. Second, no imputation was performed, and no statistical correction, such as inverse probability weighting, was applied to address potential bias due to loss to follow-up. However, it is worth noting that, given the high retention rate and the use of a single standardized time point, potential bias from missing data is likely to be limited. Third, we did not perform an analysis of the main outcomes among those who answered the messages. However, responding to SMS text messages remained relatively stable over the 48 weeks, with values varying slightly around 70%, suggesting consistent exposure and passive engagement with the intervention (Figure 3). Fourth, we did not capture participant engagement behavior, such as response latency, SMS text message read rate, or interaction dynamics over time. Fifth, self-reported adherence is subjected to recall, response, or social desirability bias [42,43]. Sixth, our study was conducted in 3 sites in Rio de Janeiro and São Paulo and thus cannot be generalized to all Brazilian MSM and transgender women. Seventh, only 6% of the overall sample was composed of transgender women. Finally, other apps designed to support medication adherence are available in Brazil, which could have interfered in our analysis.

### Conclusions

Weekly SMS text message reminders successfully improved PrEP adherence among young MSM in Brazil. Future interventions should consider leveraging more widely adopted mHealth tools, such as WhatsApp or dedicated adherence apps, to better support PrEP use among MSM and transgender women in Brazil.

## Supplementary material

10.2196/72360 Checklist 1 CONSORT-EHEALTH (Consolidated Standards of Reporting Trials of Electronic and Mobile Health Applications and Online Telehealth) checklist.

## Abbreviations

CONSORT-EHEALTH: Consolidated Standards of Reporting Trials of Electronic and Mobile Health Applications and Online Telehealth
ED-PrEP: event-driven oral PrEP
mHealth: mobile health
MPR: medication possession ratio
MSM: men who have sex with men
PrEP: pre-exposure prophylaxis
SOC: standard of care
TFV-DP: tenofovir-diphosphate
